# Comparison of the external load of professional goalkeepers in different weekly training sessions

**DOI:** 10.5114/biolsport.2024.129484

**Published:** 2023-10-04

**Authors:** David Casamichana, Eider Barba, Andres Martín-García, Iñaki Ulloa, Fabio Yuzo Nakamura, Julen Castellano

**Affiliations:** 1 Real Sociedad de Fútbol, Donostia-San Sebastián, Spain; 2 University of the Basque Country (UPV/EHU), Vitoria-Gasteiz, Spain; 3 Society, Sports and Physical Exercise Research Group (GIKAFIT), Spain; 4 FC Barcelona, Barcelona, Spain; 5 Research Center in Sports Sciences, Health Sciences and Human Development (CIDESD), University of Maia, Maia, Portugal

**Keywords:** Football, GPS, Periodization, Training load, Microcycle

## Abstract

The aim of this study was to compare the external load of professional goalkeepers (GKs) in different training sessions of the microcycle. Three professional GKs (age: 28.1 ± 6.9 years; stature: 190.1 ± 1.9 cm; body mass: 84.8 ± 1.1 kg) were monitored by GPS devices during different training sessions according to the days since/until the match day (MD) at MD+1, MD-4, MD-3, MD-2 and MD-1. Different external load measures were calculated: total distance, distance covered at > 14 km · h^−1^, acceleration load, player load, number of dives, dive load, number of explosive efforts of displacement, number of low intensity (< 0.3 m), medium intensity (0.3–0.4 m), high intensity (> 0.4 m) and medium-high intensity jumps (> 0.3 m). The results showed that there is a decrease in the external load as the GKs’ training sessions approach the match, with the lowest value of external load observed at MD-1, and the highest external loads at MD+1 and MD-4. This analysis of the external load demands of professional soccer GKs provides new information that will be useful to inform professionals when planning and implementing training and/or recovery strategies for soccer GKs during the microcycle.

## INTRODUCTION

During a soccer match, one of the goalkeeper’s (GK) primary roles is to protect their team’s goal, performing multiple individual actions that directly influence the outcome of a match [1]. It is a very different role compared to the rest of the players due to the possibility of touching the ball with the hands, and the specific positioning on the field, with consequences of errors very directly related to the match outcome [2, 3]. Other important functions of GKs include delivery of the ball during the initiation of an attack and taking control of the field of play [1]. The motor actions performed by these players are generally explosive and of short duration, and include diving, catching, jumping, throwing and explosive accelerations/decelerations [4].

Several studies have quantified the activity profile of GKs during soccer matches [2, 5, 6]. GKs cover a distance during a match that is close to 50% of that covered by outfield players [5], comprising only a few short sprints, typically of very short distance [4, 5, 7]. Considering that a GK plays in a limited space, it is more difficult for him/her to reach a large number of high-velocity actions [8, 9]. However, GKs’ high-intensity actions are highly decisive in a match, especially the explosive actions performed in short distances between 0 and 5 m, which are the most prevalent during competition [5]. In the Spanish League, the distances covered between the 2011–2012 and 2016–2017 seasons did not significantly change among GKs, but players in the teams at the bottom of the ranking covered longer distances at high-speed running than their peers in the top ranked teams [6]. This may be because matches between unbalanced opponents produce more open game contexts than those between balanced opponents [10]. Other studies suggest that, in the second half of matches, the number of actions performed by GKs and the number of goal opportunities/goals increase due to physical fatigue of the outfield players [4, 5]. Therefore, more dives and explosive efforts are observed among GKs during the final 15 min of the match [4].

It is extremely important to know the match demands of each player so that training programmes can be adjusted appropriately [5, 7]. In this way, it is possible to design specific exercises that take into account the physical demands placed on a GK. However, the most commonly reported external load variable in GKs is the distance covered at different ranges of speed [5, 11]. For this reason, the loads imposed by high-intensity movements with minimal changes in position (saves, clearances, slides, etc.) are usually undervalued [12]. When it comes to the GK’s training, it is important to improve players’ ability in explosive movements by combining short high-intensity exercises with sufficient rest periods [13], because the most frequent competitive actions are the save, control and clearing [14].

Nonetheless, there is very little information regarding the GK’s training load during a typical training week. Malone et al. [7] described the load imposed on a single GK as a function of the number of days until/since the match day (MD), finding that the highest values of external and internal load occur at MD-3 for most variables, while the lowest values are found close to the match (MD-1 and MD+1). Recently, Moreno-Pérez et al. [12] observed higher external load values in most of the variables investigated at MD-4, with a decrease in activity as training sessions approached the match.

However, there is little information on relevant and specific variables for GK training such as the number of explosive actions performed, the number of jumps of different intensities and dives. Therefore, the aim of this study was to compare the external load of professional GKs from an elite European club in different training sessions within the typical microcycle, according to the number of days until/since the MD.

## MATERIALS AND METHODS

### Subjects

Three professional soccer GKs (age: 28.1 ± 6.9 years; stature: 190.1 ± 1.9 cm; body mass: 84.8 ± 1.1 kg) belonging to the same Spanish First Division team participated in the study. The training sessions were carried out on a natural grass field at 11:00 a.m. All GKs were familiar with the training protocols prior to the investigation. The monitored sessions occurred between September and January of the relevant season. Only sessions belonging to weeks with a single match were included in the analyses. Finally, as an inclusion criterion, only sessions in which GKs completed all the planned training time were included. The statistical power was computed with the G*Power stand-alone power analysis program (version 3.1.9.6 for Windows, Institut für Experimentelle Psychologie, Düsseldorf, Germany). For the total sample size of 114 records, an effect size of 0.50 and an α error of probability of 0.05, the power (1–β err prob) was 0.99. Data arose as a condition of the players’ employment whereby they were assessed daily; thus no authorization was required from an institutional ethics committee [15]. Nevertheless, this study complied with the Declaration of Helsinki and was approved by the Ethics Committee of the University of the Basque Country (UPV/EHU, code: M10-2021-153).

### Measures

The external training load was collected using GK-specific 10 Hz GPS devices (Vector, Catapult innovations Ltd., Australia). Players wore the GPS devices from the beginning to the end of each training session. The GPS device was fitted to the upper back (i.e., between the shoulder blades) of each player in a specifically designed neoprene harness to minimise movement artefacts. After each training session, the data were extracted to a computer and analysed using the manufacturer’s software (OpenField, version 2.4.0 Build #55403; Catapult innovations Ltd., Australia).

The external load variables analysed were the following: total distance covered (m), distance covered at > 14 km · h^−1^ (m), player load (PL, AU), acceleration load (Aload, AU), number of dives (n), total dive load (AU), number of high intensity (> 0.4 m), medium intensity (0.3–0.4 m), low intensity (< 0.3 m) and medium-high intensity (> 0.3 m) jumps, the number of total jumps (n) and the number of explosive displacement efforts (n; combined number of high-intensity accelerations (> 3 m · s^−2^), decelerations (< -3 m · s^−2^) and change of directions to the left and right).

PL is a measure expressed in arbitrary units obtained through the sum of the accelerations across all axes of the tri-axial accelerometer embedded in the GPS units. Previous research on this indicator has reported high intra- and inter-device reliability [16], and it has been shown to be a valid metric to measure training load in soccer players [17]. The Aload is a variable calculated making all accelerations and decelerations positive, providing an indication of the total acceleration requirements of the athlete, irrespective of velocity [18]. A previous study [19] showed an inter-unit reliability of 2–3% regarding Aload, and this is lower (i.e., higher reliability) than what is typically seen between devices using more commonly used acceleration-based assessments (number of accelerations/decelerations and distance covered in different intensity thresholds). The jumping intensity thresholds were based on previous studies [4]. The dive load quantifies the “work” performed by a GK during a dive, and it is calculated as the sum of three sub-metrics: pre-dive load – the sum of all movements of the GK during the second before the impact; dive impact – the force with which the GK hits the floor; and post-dive load – the sum of all movements of the GK during the second after impact. Hence, dive load = pre-dive load + dive impact + post-dive load.

It was previously reported that the ability of the GPS (and the inertial measurement unit) devices to detect GK-specific action counts was very high, with a nearly perfect correlation (r = 0.903) with the number of counts via video analysis, the accuracy to detect dives being better than that to detect jumps [20]. Although jump height presents high test-retest reliability (intra-class correlation coefficient = 0.86–0.88), it displays mean bias of -2.74 cm when compared to the criterion 3D motion analysis system [21].

The number of satellites used to infer GPS signal quality, horizontal dilution of precision and the average of the GNSS quality were 11.7 ± 0.8 satellites, 0.8 ± 0.1 and 66.8 ± 9.7 %, respectively.

### Procedures

The study was conducted during the 2019–2020 competitive season and a total of 114 individual GPS files from training sessions were analysed. All training sessions performed on the field were monitored using GPS devices. They were activated according to the manufacturer’s guidelines ~40 min before commencing each training session and players wore the same device throughout the study in order to avoid inter-unit variability.

The microcycle was adjusted to the players’ competitive schedule, the state of physical recovery and the conditioning requirements. This study analysed training weeks where players had 6 days between successive matches, and the training week comprised 5 training sessions that clearly focused on the upcoming match [22]. Training load data were analysed with respect to the number of days before or after a match (MD minus or plus [MD- or MD+] approach) [22, 23]. The training sessions of a typical microcycle described below encompass specific contents for GKs, combined with exercises with outfield players:

MD+1: GKs combined training with players who had completed less than 60 minutes in the previous match. In this training session, work was carried out within a technical circuit followed by a positional game and a small-sided game with GKs (area: 30–60 m^2^ per player), with exercises where outfield players developed maximal speed and high-speed running and finishing.

MD-4: GKs warmed up with the goalkeeper coach and after that, the training aimed to develop the strength and power capabilities of the players. This session consisted of gym training followed by positional games and a small-sided game with GKs (area: 25–50 m^2^ per player). The former comprised intensive exercise with a low number of players per team, small pitch dimensions per player and shorter repetitions.

MD-3: After warming up with the goalkeeper coach, GKs performed an extensive training session, with more players per team, larger pitch dimensions per player and longer repetitions. The structure consisted of specific GK exercises with positional games (area: 70–100 m^2^), finishing the session with an 11 vs. 11 game (72 × 65 m).

MD-2: This session aimed to tactically prepare the players for the next match. This session finishes with finishing drills.

MD-1: GKs warmed up with the goalkeeper coach and, after that, they participated with the rest of the outfield players in drills replicating the tactical competition scenarios and concluded with set pieces.

### Statistical Analysis

The descriptive statistics were calculated and reported as mean ± standard deviation of the mean (SD) for each training session relative to MD, for each variable. The differences between the independent variable MD (− or +) training session in all measured variables (dependent variable) were examined using repeated measures analysis of variance (ANOVA). Post hoc analyses were performed using Bonferroni’s honestly significant difference test. Differences between training sessions were assessed via standardized mean differences (Cohen’s d with 90% confidence limits). The interpretation thresholds for standardized effect size (ES) were as follows [24]: < 0.2 (trivial), 0.2–0.6 (small), 0.6–1.2 (moderate), 1.2–2.0 (large), and > 2.0 (very large). JASP version 0.7.5 was used to conduct the analysis and statistical significance was set at p < 0.05.

## RESULTS

Table 1 presents the absolute external load variables obtained by GKs in training sessions according to the days before/after the MD. Regarding global indicators, MD-4 displayed higher values than MD-3, MD-2 and MD-1 in Aload, TD and PL (effect size, ES ranged from 0.3 to 1.6). MD+1 showed the highest load in the number of explosive efforts of displacement, and number of low, medium and medium-high intensity jumps (ES: 0.2–1.2), while MD-3 resulted in the highest number of high intensity jumps (> 0.4 m) with respect to the rest of the training sessions (ES: 0.01–0.4). Furthermore, a greater distance covered at > 14 km · h^−1^ at MD-3 was observed (ES: 0.2–0.4), while sessions MD+1 and MD-4 had a higher dive load (ES: 0.5–1.3).

**TABLE 1:** External load metrics (mean ± SD) according to the training session time since/until match day.

Training days	Total distance (m)	Distance > 14 km/h (m)	Player load (AU)	Acceleration load (AU)	Dive Load (AU)	Low intensity jumps (< 0.3 m; n)	Medium intensity jumps (0.3–0.4 m; n)	High intensity jumps (> 0,4 m; n)	Medium-high intensity Jumps (> 0.3 m; n)	Explosive efforts of displacement (n)
MD+1	3221.4 ± 628.9	44.7 ± 38.2	369.1 ± 78.8^b^	1695.2 ± 333.0^b^	438.6 ± 148.0^a,b^	19.7 ± 9.9^a,c^	11.0 ± 10.1^d^	4.1 ± 4.6	15.1 ± 12.1	53.8 ± 17.5^a,b,c^
MD-4	3611.3 ± 1141.7^b^	30.0 ± 21.8	377.2 ± 120.4^b^	1846.4 ± 631.5^b,c^	453.1 ± 215.4^a,b^	14.3 ± 8.9	4.3 ± 3.6	4.5 ± 5.1	8.8 ± 7.4	49.2 ± 15.4^a,b,c^
MD-3	3296.4 ± 484.9^b^	49.9 ± 43.5	318.9 ± 44.4	1415.6 ± 206.5	245.3 ± 132.0	10.0 ± 6.3	6.5 ± 6.4	5.0 ± 4.6	11.4 ± 9.6	28.5 ± 14.2
MD-2	2958.2 ± 738.9	37.7 ± 33.6	322.3 ± 71.6	1376.1 ± 335.4	316.0 ± 101.6	11.6 ± 6.1	6.7 ± 4.8	4.0 ± 4.6	10.7 ± 7.1	35.3 ± 12.4
MD-1	2654.3 ± 635.8	36.2 ± 32.7	294.8 ± 69.7	1185.5 ± 306.9	256.7 ± 125.1	12.9 ± 9.4	6.6 ± 5.7	3.7 ± 3.0	10.3 ± 7.5	31.2 ± 14.0
ES; p:	ES: 0.3–1.2; p < 0.001	ES: 0.0–0.4; p = 0.557	ES: 0.3–1.2; p = 0.002	ES: 0.5–1.6; p < 0.001	ES: 0.1–1.3; p < 0.001	ES: 0.1–1.2; p = 0.008	ES: 0.01–0.9; p = 0.045	ES: 0.01–0.4; p = 0.858 p	ES: 0.1–0.6; = 0.286	ES: 0.2–1.6; p < 0.001

Note: MD is match day; MD+1: is the training session 1 day after the previous match; MD-4 is the training session 4 days before the next match; MD-3 is the training session 3 days before the next match; MD-2 is the training session 2 days before the next match; MD-1: is the training session 1 day before the next match;

a > MD-3;

b > MD-1;

c > MD-2;

d > MD-4.

**FIG. 1:**
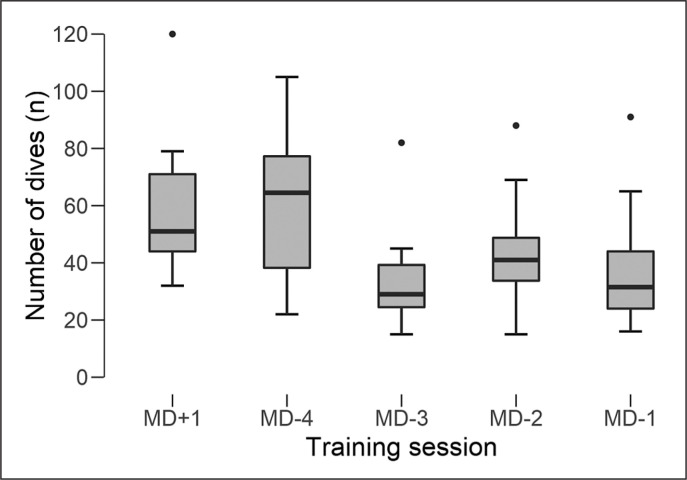
Number of dives according to the training session time since/until match day. Note: MD is match day; MD+1: is the training session 1 day after the previous match; MD-4 is the training session 4 days before the next match; MD-3 is the training session 3 days before the next match; MD-2 is the training session 2 days before the next match; MD-1: is the training session 1 day before the next match; ●: higher number of dives than MD+1, MD-3, MD-2 and MD-1 (p < 0.001).

Figure 1 shows the absolute number of dives that GKs performed across the training sessions. At MD+1 and MD-4, a higher number of dives was observed compared to MD-3, MD-2 and MD-1 (ES: 0.4–1.3).

## DISCUSSION

The purpose of this study was to investigate the weekly external load dynamics of professional soccer GKs. The main findings of the study were that there was a decrease in the level of external load as the GK approached the match, with the lowest value of external load at MD-1 and the highest level of external load at MD+1 and MD-4.

There is little information in the literature regarding the weekly load dynamics of GKs in professional soccer. However, there seems to be a tendency to reduce the load as training sessions approach the game, similarly to what is observed in outfield players [22]. In our study, the highest load levels were found at MD-4, in agreement with the observations of Moreno-Pérez et al. [12]. However, Malone et al. [7] found that the highest values of training metrics were observed at the MD-3 session. Different training methodologies among clubs may explain the differences in results. Teams that plan training according to tactical periodization will place situations in small pitches at MD-4 [25, 26]. These training situations with a low number of players per team and small pitch dimensions increase the frequency of shooting/finishing [27]; therefore, they can increase the external load imposed on GKs during the session. However, this type of activity is characterized by very little aerial play, which could lead to a reduced average number of jumps [4], this being the main reason for MD-4 attaining the lowest load of the week in the number of medium and medium-high intensity jumps.

The tapering strategy has proven to be effective in increasing match performance [28]. This decline in training load as the match gets closer seems to be a strategy adopted by soccer teams frequently, with the intention of promoting recovery processes before the match [22, 29]. In the case of GKs, previous studies have shown that the lowest external load of the microcycle is at MD-2 [12] or at MD-1 [7]. In our study, there were no significant differences between the two sessions in any of the load variables analysed, but the MD-1 session tended to have a lower external load than the MD-2.

On the other hand, according to the scientific literature, the MD+1 session focuses on the active recovery of GKs [7, 12]. Nevertheless, in our case it seems that the MD+1 session had a similar external load as the MD-4 session, having higher values in some variables and lower in others, with a significant difference in the number of medium intensity jumps (0.3–0.4 m). In our study, three GKs from the same team participated, so only one of the three had played the previous match. In this case, the other two GKs do not need an active recovery session and hence they perform a compensatory training session, this being a possible reason for them displaying an external load similar to MD-4. In addition, a small number of players usually participate in this session [30], which could explain this similarity of loads between MD+1 and MD-4.

In combination with other studies, there is a pattern of total distance covered throughout the microcycle. In our study, the lowest total distance values were found at MD-1, approximately 2600 m, and the highest values at MD-4, approximately 3600 m, being similar to the values reported by Malone et al. [7] and Moreno-Pérez et al. [12]. It is also clear that GKs cover a greater total distance in the match than in training sessions [5, 7, 11].

Given that specific performance variables are missing for GKs and that there is a lack of information that characterizes the type of training performed, it is difficult to make direct comparisons of the other variables between the different studies. Nevertheless, White et al. [4] found that GKs made approximately 51 dives in training sessions between matches. In our study, there were sessions that accumulated between about 30 and 60 dives, which means that the results are similar in both studies. According to White et al. [4], GKs make about 10 dives during a match, with the number of dives being significantly higher throughout the training week. On the other hand, very similar values of PL can be seen in the MD-4 session of the Malone et al. [7] study and in our study (approximately 350–400 AU). It is known that a GK has an external load of about 550 AU on the PL variable during a match [7], so it can be inferred that in our study GKs showed a lower PL in the MD-4 session than in the matches. Nonetheless, to confirm these inferences, it would be interesting to make an analysis of the match demands in our GKs. It would also be interesting to know the circumstances of the matches, as GKs of teams in the lower zone of the ranking are generally subjected to a higher external load. Future studies concurrently analysing GPS-derived measures and video analysis can help clarify this topic.

The present study has several limitations that should be taken into account. Firstly, only three GKs belonging to a single professional soccer team were monitored. Therefore, the external load distribution in each type of session will be directly affected by the specific load management carried out by the team analysed. In this sense, information about external load management in other professional soccer teams is required. Secondly, completing the external load values with information on internal load or technical-tactical aspects would be relevant in order to know the demands linked to the different types of sessions analysed. Finally, completing information on the external and internal load demanded of goalkeepers with the contextualization of the features of the tasks performed would provide a more practical proposal that goalkeeping coaches could use in the design of training tasks.

## CONCLUSIONS

This analysis of the external load demands of professional soccer GKs provides new information that will be useful to inform professionals when planning the preparation and implementation of training and/or recovery strategies for soccer GKs during the course of the competitive microcycle. This study shows that the goalkeepers’ load management presents an external load reduction as they get closer to the official match, with the lowest value of external load at MD-1. The first two post-match sessions accumulate the highest external load levels.
